# Downsizing chronic disease management programs for type 2 diabetes patients during the COVID-19 pandemic: changes in healthcare utilization patterns

**DOI:** 10.3389/fmed.2025.1490175

**Published:** 2025-06-11

**Authors:** Corinne Rijpkema, Lotte Ramerman, Lilian Peters, Jean Muris, Tim Olde Hartman, Maarten Homburg, Isabelle Bos, Robert Verheij

**Affiliations:** ^1^Health Data and Learning Health Systems, Nivel Netherlands Institute for Health Services Research, Utrecht, Netherlands; ^2^Tilburg School of Social and Behavioral Sciences, Tilburg University, Tilburg, Netherlands; ^3^Department of Primary and Long-Term Care, University Medical Centre Groningen, University of Groningen, Groningen, Netherlands; ^4^Midwifery Science, AVAG, Amsterdam Public Health, Amsterdam University Medical Centre, Vrije Universiteit Amsterdam, Amsterdam, Netherlands; ^5^Department of Family Medicine, CAPHRI Care and Public Health Research Institute, Maastricht University, Maastricht, Netherlands; ^6^Department of Primary and Community Care, Radboud University Nijmegen Medical Centre, Radboud Institute of Health Sciences, Nijmegen, Netherlands; ^7^National Health Care Institute, Diemen, Netherlands

**Keywords:** diabetes mellitus type 2, general practice, COVID-19, electronic health records, hospital care, secondary prevention

## Abstract

**Background:**

During the COVID-19 pandemic, chronic disease management programs (CDMP) for Dutch type 2 diabetes patients by general practitioners (GP) were scaled down. These programs aim to improve diabetes prognosis through appropriate interventions and avoid hospital treatment. However, it remains unknown whether downsizing CDMP increased care in other settings. Therefore, we examined the changes in healthcare utilization for type 2 diabetes patients during the COVID-19 pandemic including CDMP, GP out-of-hours care, hospital care, and regular GP care.

**Methods:**

Routine healthcare data from electronic patient records of GPs, participating in Nivel Primary Care Database, of 15,247 Dutch type 2 diabetes patients enrolled in CDMP, were linked to GP out-of-hours registration data and hospital claims data. Regression analyses compared healthcare utilization in 2020 and 2021 (pandemic) to 2019 (non-pandemic).

**Results:**

For most quarters of 2020 and 2021, care through CDMP was significantly lower, down to 38% in Q2 of 2020 compared to 2019. In Q1 of 2020, type 2 diabetes patient visits to out-of-hours GP services rose notably, but decreased in Q1 of 2021, compared to 2019. Hospital care for diabetes showed a significant increase in Q2 of 2021 (+11.3%), compared to Q2 2019 and regular GP care increased from Q1 2021 (up to +11.1% in Q3 2021). Although no significant differences were observed in other quarters, there were different trends visible. Reduced CDMP contacts in 2020 were significantly associated with increased regular GP care in 2021. Moreover, reduced CDMP in early 2021 was significantly associated with more regular GP care and hospital care later in 2021.

**Conclusion:**

Downscaling CDMP care for type 2 diabetes patients during the COVID-19 pandemic was associated with temporary increases in hospital care for diabetes and regular GP care at various times during the pandemic. These findings may contribute to making informed decisions regarding measures during future pandemics, and, therefore, the pandemic provided a unique learning opportunity for the healthcare system in delivering appropriate care through CDMP. In future pandemics, it will be essential to implement adaptations such as telemedicine to mitigate health deterioration and alleviate pressure on other healthcare services.

## 1 Introduction

The COVID-19 pandemic has had a massive impact on public health, as evidenced by the number of reported COVID-19 cases and deaths (1). Consequently care, including chronic disease management programs (CDMP) at general practitioners (GPs), was downscaled, both by GPs to prevent the spread of the virus and by patients out of fear of contracting COVID-19 (2, 3). This may have had major consequences for individuals with chronic conditions such as diabetes mellitus.

By 2021, 1.1 million people in the Netherlands suffered from diabetes (4), ranking third in terms of disease burden (DALY) (5). In the Netherlands, GPs act as the first health contact and gatekeepers to specialized hospital care (6). They also play a central role in CDMP, alongside practice nurses who address relatively more non-complex somatic and mental health problems (7). Approximately 500,000 type 2 diabetes patients participate in CDMP, offered by their GP, practice nurse, dietitian, and other paramedics where disease burden is assessed, medication, and lifestyle are discussed, self-management is encouraged and any indication for referral to other healthcare providers is assessed (8, 9). These regular check-ups are intended for early detection, to reduce symptoms and prevent worsening of the disease, as long-term uncontrolled type 2 diabetes, for example, can lead to permanent vascular damage, diabetic retinopathy or diabetic neuropathy (9). Patients are included in a CDMP if diagnosed with type 2 diabetes mellitus and aged 18 years or older (10). Patient's willingness and motivation to participate are also assessed beforehand (10). Pregnant women and women planning a pregnancy, women with gestational diabetes, individuals with diabetes in remission without glucose-lowering medication, patients with type 1 diabetes or those already participating in a care program for frail elderly are not eligible for CDMP (10). The costs of CDMP are fully covered by the health insurance company and is not subject to the patient's deductible. All Dutch citizens are required to have basic health insurance, which includes coverage for general practice care (6). In addition, CDMP is part of bundled payment, meaning that individual consultations provided as part of CDMP cannot be claimed separately.

During the pandemic, type 2 diabetes patients were affected by both the downscaling of care through CDMP and social constraints. The scaling down of CDMP, such as reduced self-management support and education for type 2 diabetes patients by healthcare providers (11), as well as becoming ill due to COVID-19, can worsen outcomes for type 2 diabetes patients, e.g., glucose variability, hospitalization or death (12, 13). Moreover, social constraints during the COVID-19 pandemic can lead to psychological problems, including anger, confusion, and Post Traumatic Stress Disorder (PTSD)-like symptoms (14), which affect disease symptoms as eating habits change, physical activity decreases, and medication adherence decreases (12, 15). Both the downscaling of care through CDMP and social constraints may, in turn, have led to worsening and deterioration of their disease and increased need and care utilization, requiring (unplanned) care from other healthcare professionals, such as GP out-of-hours services, emergency departments, or hospitals (16, 17). CDMPs also act as a safety net for individuals by addressing changes in eating habits, decreased physical activity, and medication adherence. When this care is no longer available, there is an increased risk that these individuals will become destabilized, leading to a greater need for care from other healthcare professionals (17). This is all the more true because social constraints during the pandemic limited the support of patients' social networks. Such shifts in healthcare utilization offer insight into the consequences of scaling down CDMP for type 2 diabetes patients within GP care, making the pandemic a unique learning opportunity for providing appropriate care.

Therefore, this study aimed to provide insight into the changes in contact rates for type 2 diabetes patients in 2020 and 2021 compared to 2019 with regard to (1) CDMP consultations with GPs, (2) care through out-of-hours GP services, (3) hospital care for diabetes, and (4) regular care by GP practices. Analyzing the impact on healthcare utilization after scaling down CDPM could provide valuable insights into the post-pandemic effectiveness of these programs, especially considering the current challenges facing GP care, such as increased demand for care due to an aging population, task shifting to GPs, and staff shortages (7, 18, 19).

## 2 Materials and methods

### 2.1 Study design and data sources

For this retrospective observational study, we used existing data. These data were derived from the Nivel Primary Care Database (Nivel-PCD), which contains deidentified data from electronic health records (EHR) from GP practices (~500, representing 10% of the Dutch population) and out-of-hours (OOH) GP services (60% of services, representing a joint catchment area of almost 12.3 million people from the Netherlands) (20, 21). Both data sources include the number of contacts, types of contact, health problems presented during these contacts entered with International Classification of Primary Care 1 (ICPC-1) and the insurance claims associated with these. Data from GP practices also include results of diagnostic tests requested by GPs. Data from Nivel-PCD were linked at patient level to Microdata from Statistics Netherlands (CBS), an organization tasked with collecting, processing and publishing statistics for the benefit of practice, policy and science. The pseudonymized CBS Microdata contains data on the Dutch population, health and wellbeing, income and also include medical specialist claims data (obtained via Vektis). This study follows the STROBE statement (22).

### 2.2 Patient selection

We used data from patients (a) aged 18 years and older, (b) with an active diagnosis of diabetes recorded in 2019 or before, (c) registered in a GP practice participating in Nivel-PCD, (d) residing in the catchment area of an OOH GP service that also participated in Nivel-PCD, (e) for three consecutive years (2019–2021) and (f) enrolled in CDMP for type 2 diabetes. Care contacts through CDMP cannot be inferred from the claims dataset, as such care contacts cannot be claimed. Relying solely on the claim for “enrollment in the CDMP” does not provide confirmation that individuals received care. For the purposes of this analysis, it was essential to ensure that patients had received care in 2019. Therefore, we selected patients using the following criteria:

1. Recording in the EHR for diabetes, using ICPC1-code: T90 (23).
2. At least one recorded outcome from (diagnostic) testing that is part of the CDMP, such as glucose levels or blood pressure.

*Exclusion:* a declared GP consultation for diabetes to a health insurer. CDMP is part of bundled payment and therefore individual consultations as part of CDMP are not allowed as individual claims. These are considered regular GP consultation, not part of the CDMP.

### 2.3 Variables

The primary outcomes of this study were the changes in contact rates in CDMP, hospital care for diabetes, care at OOH GP services and regular GP care for both diabetes and other diseases, expressed as percentage change, as the number of contacts per 1,000 patients or as difference scores. CDMP contact rates were determined based on the two criteria mentioned in section “patient selection.” Hospital care for diabetes was based on claims data of medical specialist care, see Supplementary Table 1 for the reimbursement codes. Additionally, OOH GP services and regular GP care were based on EHR data, using reimbursed consultations for all ICPC codes, including diabetes (T90) and other health problems. Determinants were gender, age, migration background, and household income (Supplementary Table 2).

### 2.4 Data analysis

Patient characteristics were presented for 2019–2021 in absolute numbers and percentages, for all determinants. Mean (SD) contact rates for CDMP consultations, OOH GP care, regular GP care, and hospital care, were calculated per quarter in 2019–2021, along with the percentage changes for each quarter of 2020 and 2021, compared to same quarter in 2019. The percentage changes were calculated based on the mean contacts for all patients in each quarter. Quarterly analyses were conducted to reflect the different phases of the pandemic, ranging from periods of strict measures, such as lockdowns, to phases with fewer restrictions. Generalized linear regression analysis, adjusted for time series autocorrelation (weeks), assessed changes in care utilization for CDMP consultations, OOH GP care, regular GP care, and hospital care, with an interaction-term between quarter and year. A sensitivity analysis was performed for different subgroups (ages 18–64 vs. 65+, Dutch vs. migration background and low vs. middle vs. high household incomes), to address population heterogeneity. For all subgroups, contact rates per 1,000 patients for 2019, 2020, and 2021 were calculated, as well as the differences between 2020 and 2019 and between 2021 and 2019. *Long-term* (1 year later) and *short-term* (6 months later) associations of downscaled CDMP were examined using linear regression analyses to determine if differences in care through CDMP (e.g., in 2020 compared to 2019) influenced the difference in care utilization at other healthcare settings (e.g., in 2021 compared to 2019), separately for OOH GP care, regular GP care, and hospital care (adjusted for all determinants). For both analyses, we examined assumptions, including linearity, multicollinearity, normality of residuals, homoscedasticity, and the presence of outliers. These assumption checks revealed no substantial violations of the underlying assumptions. All analyses were two-tailed, with a significance threshold of 0.05. STATA software (version 16.1) was used for analysis.

## 3 Results

In total, 15,247 type 2 diabetes patients participating in CDMP were included in 2019 and followed in 2020 and 2021, 53.2% were male and 46.8% were female. Most patients were 65 years and older (62.1–67.9%), Dutch (72.8%), and had a low (49.6–52.7%) or middle (35.4–37%) household income (Table 1). Because the study followed individuals over time, there were relatively more individuals in the older age categories (75–84 and 85+) in 2021 compared to 2019. The total number of CDMP contacts decreased from 35,582 contacts for all patients in 2019 to 24,173 in 2021. OOH GP contacts initially increased in 2020 to 4,125 contacts, then declined to 3,480 in 2021, compared to 3,817 in 2019. Meanwhile, regular GP care contacts and hospital contacts initially decreased in 2020 (114,519 and 2,151 contacts, respectively), before increasing in 2021 (126,942 and 2,452 contacts, respectively), compared to 2019 (116,012 and 2,267).

**Table 1:** Characteristics of included type 2 diabetes patients.

**Characteristic**	**Type 2 diabetes patients (*****n*** = **15,247)**		
	**2019**	**2020**	**2021**
**Gender**			
Men	8,114 (53.2%)		
Women	7,133 (46.8%)		
**Age (in categories)**			
18–44	409 (2.7%)	349 (2.3%)	302 (2.0%)
45–64	5,383 (35.3%)	4,994 (32.8%)	4,594 (30.1%)
65–74	5,162 (33.9%)	5,152 (33.8%)	4,997 (32.8%)
75–84	3,486 (22.9%)	3,746 (24.6%)	4,116 (27.0%)
85 years and older	807 (5.3%)	1,006 (6.6%)	1,238 (8.1%)
**Migration background**			
Dutch	11,102 (72.8%)		
Western	2,766 (18.1%)		
Non-western	1,279 (9.0%)		
**Household income**			
Low	7,563 (49.6%)	7,767 (50.9%)	8,033 (52.7%)
Middle	5,634 (37.0%)	5,519 (36.2%)	5,394 (35.4%)
High	1,925 (12.6%)	1,815 (11.9%)	1,657 (10.9%)
Missing	125 (0.8%)	146 (0.96%)	163 (1.0%)
**Healthcare utilization (total number of contacts)**			
Diabetes care programs at the GP	35,582	26,988	24,173
Out-of-hours general practice services	3,817	4,125	3,480
Regular general practice care	116,012	114,519	126,942
Hospital care	2,267	2,151	2,452

### 3.1 Healthcare use for type 2 diabetes patients

**Figure 1:**
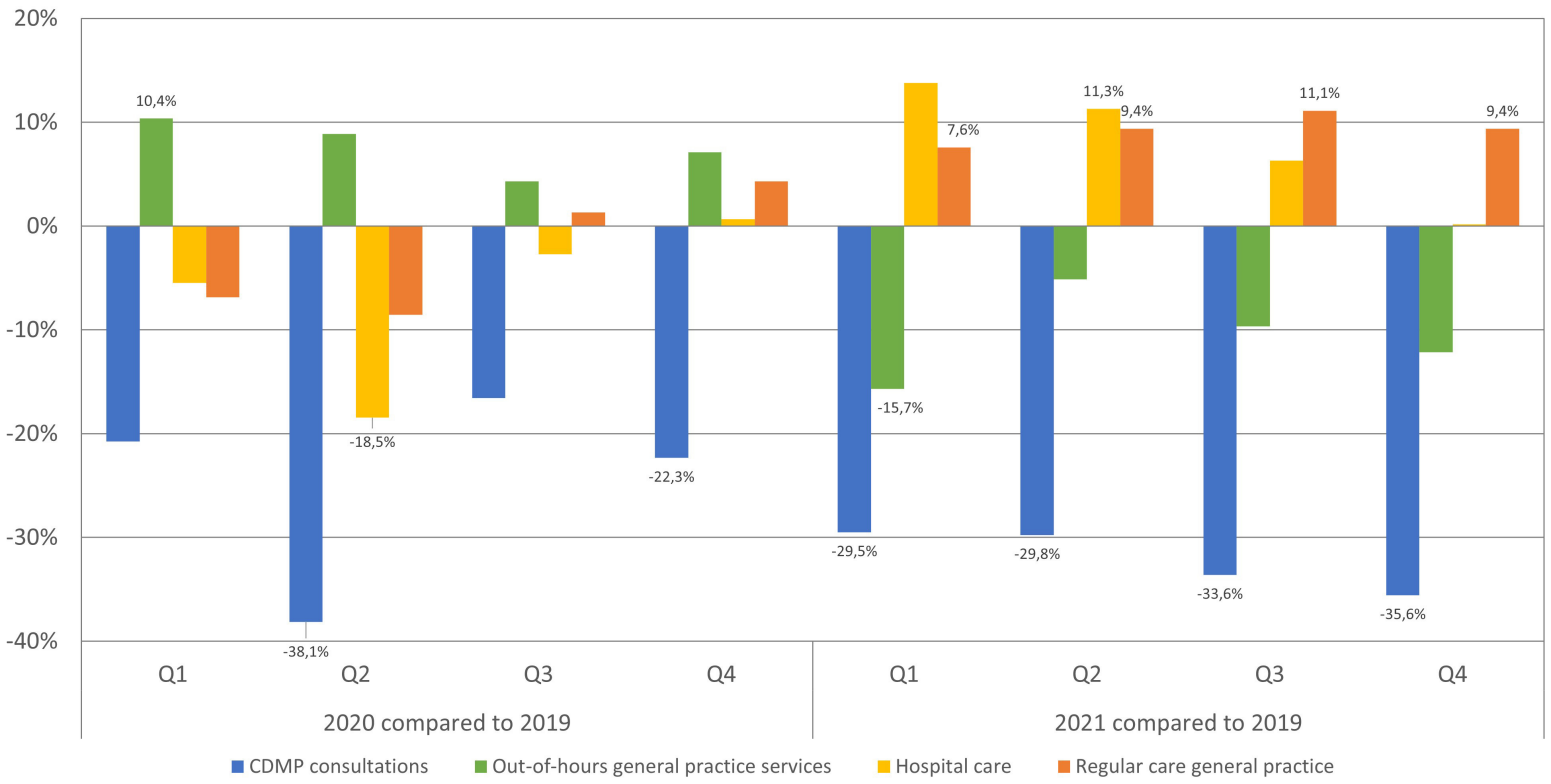
Percentage changes in healthcare utilization shown by quarter for 2020 and 2021, compared to 2019, for patients with diabetes enrolled in diabetes care programs across various healthcare settings.

During the COVID-19 pandemic, there was a notable reduction in the number of contacts taking place as part of CDMP across all quarters of 2020 and 2021, compared to 2019 (Figure 1). The most substantial decrease was observed in Q2 of 2020 (early phase of the COVID-19 pandemic), with a decline of 38.1% compared to the same quarter in 2019 (Figure 1). Both the second (Coeff: −17.0, 95% CI: −27.7 to −6.2, *p* = 0.002) and fourth (Coeff: −10.1, 95% CI: −16.6 to −3.5, *p* = 0.003) quarters of 2020, along with all quarters of 2021, showed significantly lower contact rates in CDMP than 2019 (Table 2).

**Table 2:** Mean standard deviation, and percentage change compared to 2019 for the contact rates per 1,000 type 2 diabetes patients in 2019, 2020 and 2021, and differences in contact rates between (2020 and 2019) and (2021 and 2019) tested for various healthcare settings.

		**2019^b^**	**2020**	**2021**	**Difference between 2020 and 2019^b^**				**Difference between 2021 and 2019^b^**			
**Type of care**	**Quarter**	**Mean no. of consults (SD)**	**Mean no. of consults (SD)** ***% change compared to 2019***	**Mean no. of consults (SD)** ***% change compared to 2019***	**Coefficient**	**95% CI**		***p*** **-Value**	**Coefficient**	**95% CI**		***p*** **-Value**
Diabetes care programs at the GP	Q1	47.3 (10.7)	37.4(15.4) *−20.8%*	33.3 (4.4) *−29.5%*	−9.81	−20.7	1.1	0.078	−13.9	−20.0	−7.9	<0.001^a^
	Q2	44.5 (8.6)	27.5(13.7) *−38.1%*	31.2 (6.2) *−29.8%*	−17.0	−27.7	−6.2	0.002^a^	−13.2	−19.1	−7.4	<0.001^a^
	Q3	42.7 (8.6)	35.7(6.4) *−16.6%*	28.4 (6.5) *−33.6%*	−7.1	−14.7	0.6	0.070	−14.4	−22.1	−6.7	<0.001^a^
	Q4	45.0 (10.6)	35.0(7.6) *−22.3%*	29.1 (6.5) *−35.6%*	−10.1	−16.6	−3.5	0.003^a^	−16.0	−22.4	−9.6	<0.001^a^
Out-of-hours general practice services	Q1	4.7 (0.6)	5.2(0.6) *10.4%*	4.0 (0.5) *−15.7%*	0.5	0.1	1.0	0.049^a^	−0.7	−1.1	−0.4	<0.001^a^
	Q2	4.8 (0.9)	5.3(0.7) *8.9%*	4.6 (0.5) *−5.1%*	0.4	−0.2	1.0	0.159	−0.2	−0.9	0.4	0.430
	Q3	4.8 (0.8)	5.0(0.4) *4.3%*	4.3 (0.6) *−9.7%*	0.2	−0.3	0.7	0.388	−0.5	−1.0	0.1	0.075
	Q4	4.9 (1.1)	5.2(0.9) *7.1%*	4.3 (0.9) *−12.2%*	0.3	−0.4	1.1	0.374	−0.6	−1.3	0.1	0.085
Hospital care	Q1	2.9 (0.5)	2.7(0.8) *−5.5%*	3.2 (0.5) *13.8%*	−0.2	−0.7	0.4	0.595	0.4	−0.1	0.8	0.057
	Q2	2.8 (0.6)	2.3(0.6) *−18.5%*	3.1 (0.6) *11.3%*	−0.5	−0.9	−0.1	0.011^a^	0.3	0.1	0.6	0.047^a^
	Q3	2.8 (0.6)	2.7(0.4) *−2.7%*	3.0 (0.5) *6.3%*	−0.1	−0.4	0.3	0.655	0.2	−0.2	0.5	0.334
	Q4	3.0 (0.6)	3.0(0.5) *0.7%*	3.0 (0.5) *0.2%*	0.1	−0.4	0.4	0.920	0.1	−0.4	0.4	0.980
Regular general practice care	Q1	154.1 (10.5)	143.5(31.0) *−6.9%*	165.8 (11.3) *7.6%*	−10.6	−27.2	6.1	0.213	11.7	2.8	20.5	0.010^a^
	Q2	145.7 (14.6)	133.3(17.9) *−8.5%*	159.4 (17.4) *9.4%*	−12.4	−25.6	0.7	0.063	13.7	1.5	25.9	0.028^a^
	Q3	138.8 (9.8)	140.6(13.0) *1.3%*	154.2 (12.5) *11.1%*	1.8	−9.0	12.6	0.739	15.4	4.3	26.6	0.007^a^
	Q4	146.7 (20.4)	153.0(15.8) *4.3%*	160.4 (14.9) *9.4%*	6.3	−7.4	20.0	0.367	13.7	0.1	27.5	0.050^a^

a p-Value < 0.05.

b 2019 is the reference group.

For the OOH GP services, contacts increased significantly in Q1 2020 compared to the same quarter in 2019 (Coeff: 0.5, 95% CI: 0.1–1.0, *p* = 0.049), and contacts decreased significantly in Q1 2021 compared to 2019 (Coeff: −0.7, 95% CI: −1.1 to −0.4, *p* < 0.001; Table 2). Although no significant differences were observed in other quarters, there are different trends: in all quarters of 2020, patients with diabetes visited the OOH GP services more often than in 2019. In contrast, there was a decrease in 2021 compared to 2019 in all quarters (Figure 1).

Hospital care utilization showed a significant increase only in Q2 of 2021 compared to the same quarter in 2019 (Coeff: 0.3, 95% CI: 0.1–0.6, *p* = 0.047; Table 2), and a significant decreased in Q2 2020 (Coeff: −0.5, 95% CI: 0.9 to −0.1, *p* = 0.011). However, certain trends were still observed. From the first quarter of 2021, patients with diabetes received more hospital care for diabetes, with an increase up to 13.8% compared to the same quarter in 2019 (Figure 1).

Regular GP care contact rates significantly increased for all quarters in 2021 compared to the same quarters in 2019 (Table 2). However, a trend can already be observed starting in Q3 of 2020, with an increase in GP contacts for regular GP care (Figure 1).

### 3.2 Sensitivity analysis

For individuals aged 18–64 and individuals with a migration background, there was a more substantial decline in CDMP consultations during the pandemic, compared to their comparable group, see Supplementary Table 3. Low income individuals and those over 65 experienced a greater increase in regular GP care, high-income individuals and 18–64-year-olds saw a greater increase in hospital care during the pandemic compared to their comparable groups.

### 3.3 Long-term associations with downscaled CDMP

Regular GP care in 2021 was negatively associated with CDMP consultations in 2020 (Table 3). When CDMP consultations declined in 2020 compared to 2019, regular GP care for type 2 diabetes patients significantly increased in 2021 compared to 2019 (Coeff: −0.1437, 95% CI: −0.2296 to −0.0578, *p* = 0.001). There were no significant association between the difference in CDMP consultations in 2020 compared to 2019 and the difference in OOH GP or hospital care in 2021 compared to 2019 (Table 3).

**Table 3:** The association of the difference in care utilization through chronic disease management programs for type 2 diabetes patients on the difference in care utilization at out-of-hours general practice services, hospital care or regular practice 1 year later (long-term) and 6 months later (short-term), corrected for all determinants.

**Type of care**	**Years compared**	**Coefficient**	**95% CI**		***p*-Value**
**Long-term**					
Out-of-hours general practice services	2021 vs. 2020	−0.0013	−0.0114	0.0089	0.807
Hospital care	2021 vs. 2020	−0.0063	−0.0147	0.0021	0.140
Regular general practice care	2021 vs. 2020	−0.1437	−0.2296	−0.0578	0.001^a^
**Short-term**					
Out-of-hours general practice services	*2020-2 vs. 2020-1*	0.0081	−0.0019	0.0181	0.113
	*2021-1 vs. 2020-2*	0.0010	−0.0090	0.0110	0.846
	*2021-2 vs. 2021-1*	0.0079	−0.0008	0.0167	0.076
Hospital care	*2020-2 vs. 2020-1*	0.0049	−0.0008	0.0105	0.091
	*2021-1 vs. 2020-2*	0.0007	−0.0066	0.008	0.846
	*2021-2 vs. 2021-1*	−0.0069	−0.0133	−0.0004	0.037^a^
Regular general practice care	*2020-2 vs. 2020-1*	−0.0635	−0.1312	0.0042	0.066
	*2021-1 vs. 2020-2*	0.0261	−0.0506	0.1028	0.505
	*2021-2 vs. 2021-1*	−0.0776	−0.1490	−0.0063	0.033^a^

a p-Value < 0.05.

### 3.4 Short-term associations with downscaled CDMP

There was a negative association between the difference in CDMP consultations in the first half of 2021 with the difference in hospital care and regular GP care in the second half of 2021 (Table 3). When consultations from CDMP declined in the first half of 2021 compared to the first half of 2019, hospital (Coeff: −0.0069, 95% CI: −0.0133 to −0.0004, *p* = 0.037) and regular GP care (Coeff: −0.0776, 95% CI: −0.1490 to −0.0063, *p* = 0.033) for type 2 diabetes patients significantly increased in the second half of 2021 compared to the same period in 2019.

## 4 Discussion

### 4.1 Main findings

This study showed considerable changes in healthcare utilization by type 2 diabetes patients enrolled in the CDMP during the COVID-19 pandemic, compared to 2019. As expected, there was a marked decrease in the number of consultations in the CDMP, while care utilization at OOH GP services, at hospitals (specifically for diabetes) and regular GP care temporarily increased at various times during the pandemic. The timing of these increases varied depending on the type of healthcare provider. This study indicated that, in the long-term (1 year later), reduced consultations at the CDMP were associated with increased regular GP care for type 2 diabetes patients, while in the short-term (6 months later), this association was observed for both regular GP care and hospital care (specifically for diabetes) in the second half of 2021.

### 4.2 Comparison with existing literature

In our study, we observed a decrease in consultations through CDMP for type 2 diabetes patients during the COVID-19 pandemic. At the same time, we observed a temporary increase in OOH GP care during the early stages of the pandemic in Q1 2020, which then returned to 2019 levels. Hospital care experienced a temporary increase in Q2 2021, while regular GP care increased in all quarters of 2021. Most (inter)national studies also demonstrate a decrease in diabetes care (24–26), whereas another Dutch study showed an increase in regular GP contacts by type 2 diabetes patients during the first pandemic year (27). In contrast to our study, these studies focused on all types of GP care for type 2 diabetes patients. Like our study, another Dutch study found a reduction in diabetes outpatient visits among hospitals during the first year of the pandemic for both type 1 and type 2 diabetes patients (28). Research conducted in UK showed increased emergency hospital admissions for diabetes ketoacidosis among type 2 diabetes patients from the start of the pandemic until February 2021 (29). However, a systematic review by Hartmann-Boyce et al. (30) showed that the international literature is inconclusive regarding trends in emergency care and hospitalizations among patient with type 1 and type 2 diabetes. Thus, several findings in international literature are consistent with our results, such as reduced diabetes care and increased use of hospital and regular GP care, while evidence from the systematic review also highlights inconsistencies, indicating that trends in care utilization during the pandemic may have varied across healthcare settings. At the population level, differences in healthcare utilization may be limited, however, for specific groups, the downscaling of CDMP care may have had a greater impact on healthcare utilization elsewhere. In our study, we took a first step by presenting differences in healthcare utilization across various settings for specific subgroups (e.g., age, migration background, household income). Further research is needed to better understand the impact on specific subgroups and to ensure better protection for these individuals during future pandemics. Additionally, it is important to evaluate the long-term impact of reduced care in CDMP and to gain a comprehensive understanding of its consequences.

Despite the temporary increases in healthcare utilization elsewhere, we also observe that care through CDMP did not increase again in 2021. There were various reasons for the interruption of this care during the pandemic; both for the patient, including fear of contracting COVID-19 and isolation at home, and collective factors such as shortages of medical staff and the suspension of (outpatient) care (31). Previous research indicates that continuity of CDMP is important for favorable outcomes (32, 33), including fewer complications and less healthcare utilization (34). The fact that care through CDMP has not been scaled up further may have long-term effects on health outcomes and care utilization elsewhere. This suggests the importance of CDMP, and that downscaling can be harmful for type 2 diabetes patients.

The COVID-19 pandemic may also have had a positive impact on diabetes management, largely due to the increased use of telemedicine. International studies have shown that this new approach to (self-)care contributes to better glycemic control and fewer hospitalizations (35, 36). However, we were unable to explore this in our study due to a lack of necessary data. During a crisis like the pandemic, it is not always feasible to provide care in the usual way while simultaneously preventing the spread of the virus. Therefore, in future pandemics it is essential to prioritize the limited face-to-face care available to those who need it most. At the same time, it is crucial that all type 2 diabetes patients receive the attention they need. Telemedicine offers a valuable solution for more stable type 2 diabetes patients who do not require face-to-face consultations. Such adjustments are necessary to prevent deterioration in health and thereby reduce the risk of increased healthcare utilization in other parts of the system.

### 4.3 Strengths and limitations

A strength of our study was the use of multiple real-world data sources related to type 2 diabetes patient care. To the best of our knowledge, this is the first study investigating the association of downscaling CDMP on healthcare utilization across multiple healthcare sectors. By examining data from general practices, out-of-hours GP services, and hospitals, we provided a comprehensive analysis of the broader healthcare context during the pandemic. In a crisis such as the COVID-19 pandemic, it is crucial to generate knowledge quickly, and leveraging existing data plays a vital role in this process (37).

A limitation of this study was the lack of direct information on patients' health outcomes, disease severity, additional support they received or lifestyle modifications, since this information proved difficult to obtain through the existing data sources used. These data could have explained more thoroughly the shifts in healthcare utilization. However, in times of crisis such as a pandemic, collecting additional data is particularly challenging, highlighting the importance of having access to existing healthcare data that are fit for purpose in generating timely insights into the impact of the crisis.

Another limitation is that we examined associations between the use of different types of healthcare available to patients with diabetes during a period of time in which many other factors influenced healthcare use, including lockdown measures in particular. As a result, we examined the association between two variables without implying a causal relationship or direction between them. Delayed or canceled care in CDMP may not necessarily fully explain the increase in hospital care or regular GP care. Other potential explanations for the increase in hospital care or regular GP care that were not considered in our study, include patient comorbidities, increased healthcare needs due to aging, or contracting a COVID-19 infection (13, 38, 39).

Moreover, the lockdown measures at the beginning of the pandemic may have caused these individuals to increase their use of regular GP care later, as a catch-up effect for the missed care during the early stages of the pandemic due to the lockdown measures (26). Nevertheless, we observed an increase in regular GP care in Q1 2021, even though the Netherlands was still in lockdown at that time.

Additionally, the lack of a reliable specific claim code for diabetes CDMP meant that we could only identify patients indirectly. To ensure that contacts were related to CDMP care, we applied strictly defined inclusion criteria. As a result, we may have underestimated the number of patients receiving CDMP care. In the Netherlands, more than 500,000 individuals receive care through CDMP from their GP (8).

Moreover, it was not possible to identify patients using the ICPC subcode specific to type 2 diabetes (T90.02) in the available dataset. Instead, patients were selected based on their participation in the CDMP for type 2 diabetes, which strongly indicates that they have type 2 diabetes. However, it is possible that a small number of individuals with type 1 diabetes were included based on this criterion.

Finally, in our study, we included individuals based on 2019 data and followed them throughout the study period, excluding new patients from 2020 to 2021. This may have resulted in an aging study population, with their conditions worsening over time and a potential increase in hospitalization risk.

### 4.4 Implications for research and practice

Our results indicate that the reduction in care through CDMP was associated with increased regular GP care and hospital care for diabetes during the COVID-19 pandemic. This study is a first step toward pandemic preparedness, helping the government understand how to manage healthcare postponements in future pandemics. More in-depth (qualitative) analyses are needed to examine factors influencing the association between CDMP and other healthcare, such as different levels of CDMP by GP practices, patients' needs, individual disease burden (including comorbidities), quality of life and patient self-management abilities. Conducting an inventory among GPs, healthcare professionals and patients could provide valuable insights into intended and unintended consequences of healthcare utilization. Moreover, it is important to continuously monitor healthcare utilization patterns among type 2 diabetes patients who receive CDMP care and assess the mid- to long-term impact, as this has not yet been addressed in literature. Additionally, this research serves as a starting point to investigate whether CDMP are evidence based and desirable from the perspectives of patients, GPs, hospitals, and policymakers. All these considerations are essential to determine if these healthcare shifts were unwanted by type 2 diabetes patients.

## 5 Conclusion

Downscaling CDMP care for type 2 diabetes patients during the COVID-19 pandemic was associated with (temporarily) increased hospital care for diabetes and regular GP care at various times during the pandemic. It is unlikely that these shifts in care utilization were desirable in terms of quality of care, given costs and also patient preferences. These findings may contribute to making informed decisions regarding measures during future pandemics. During future pandemics, it is essential to prioritize face-to-face consultations for those most in need while ensuring ongoing support for all patients. Telemedicine offers an effective alternative for stable patients. Such adjustments help prevent deterioration in health and reduce pressure on other healthcare services. The COVID-19 pandemic provided a unique learning opportunity to investigate the impact of downsizing CDMP for type 2 diabetes patients, as it would normally be unethical to withhold care. This situation enabled us to provide valuable insights into the effectiveness of these programs after the pandemic using real world data.

## Data Availability

The data analyzed in this study is subject to the following licenses/restrictions. The data is not publicly available due to legal constraints. Access to the data requires approval from the governing bodies of Nivel-PCD, Vektis, and CBS Microdata. Requests to access these datasets should be directed to c.rijpkema@nivel.nl.

## References

[1] World Health Organization. WHO Coronavirus (COVID-19) Dashboard. (2024). Available online at: https://data.who.int/dashboards/covid19/cases (accessed June 18, 2024).

[2] Homburg M Brandenbarg D Olde Hartman T Ramerman L Beugel G Rijpkema C. Patient experiences during the COVID-19 pandemic: a qualitative study in Dutch primary care. BJGP Open. (2022) 6. 10.3399/BJGPO.2022.0038 36270671 PMC9904784

[3] Splinter MJ Velek P Kamran Ikram M Kieboom BCT Peeters RP Bindels PJE. Prevalence and determinants of healthcare avoidance during the COVID-19 pandemic: a population-based cross-sectional study. PLoS Med. (2021) 18:e1003854. 10.1371/journal.pmed.1003854 34813591 PMC8610236

[4] VZinfo. Diabetes Mellitus. (2023). Available online at: https://www.vzinfo.nl/diabetes-mellitus (accessed March 31, 2023).

[5] VZinfo. Ranglijsten | Aandoeningen op Basis van Ziektelast (in DALY's). (2024). Available online at: https://www.vzinfo.nl/ranglijsten/aandoeningen-op-basis-van-ziektelast (accessed February 20, 2024).

[6] Kroneman M Boerma W van den Berg M Groenewegen P de Jong J van Ginneken E. Netherlands: health system review. Health Syst Transit. (2016) 18:1–239. 27467715

[7] van der Horst HE de Wit N. Redefining the core values and tasks of GPs in the Netherlands (Woudschoten 2019). Br J Gen Pract. (2020) 70:38–9. 10.3399/bjgp20X707681 31879312 PMC6919494

[8] InEen. InEen Benchmark Ketenzorg 2022. (2023). Available online at: https://ineen.nl/ineen-benchmarks/ineen-benchmark-ketenzorg/ (accessed February 22, 2024).

[9] Bilo H Dankers M Rooij de A Hart H Houweling S Ijzerman R. NHG-Standaard Diabetes Mellitus Type 2 (M01). (2024).

[10] InEen. Handleiding Inclusie en Exclusie Ketenzorgprogramma's 2024. (2024).

[11] Forde R Arente L Ausili D De Backer K Due-Christensen M Epps A. The impact of the COVID-19 pandemic on people with diabetes and diabetes services: a pan-European survey of diabetes specialist nurses undertaken by the foundation of European nurses in diabetes survey consortium. Diabet Med. (2021) 38:e14498. 10.1111/dme.14498 33314244 PMC7883040

[12] Fisher L Polonsky W Asuni A Jolly Y Hessler D. The early impact of the COVID-19 pandemic on adults with type 1 or type 2 diabetes: a national cohort study. J Diabetes Complications. (2020) 34:107748. 10.1016/j.jdiacomp.2020.107748 33059981 PMC7539933

[13] Narres M Claessen H Kvitkina T Rosenbauer J Scheider M Morbach S. Hospitalisation rate and mortality among people with and without diabetes during the COVID-19 pandemic year 2020. Eur J Epidemiol. (2022) 37:587–90. 10.1007/s10654-022-00865-6 35674859 PMC9175520

[14] Brooks SK Webster RK Smith LE Woodland L Wessely S Greenberg N. The psychological impact of quarantine and how to reduce it: rapid review of the evidence. Lancet. (2020) 395:912–20. 10.1016/S0140-6736(20)30460-8 32112714 PMC7158942

[15] Ruissen MM Regeer H Landstra CP Schroijen M Jazet I Nijhoff MF. Increased stress, weight gain and less exercise in relation to glycemic control in people with type 1 and type 2 diabetes during the COVID-19 pandemic. BMJ Open Diabetes Res Care. (2021) 9:e002035. 10.1136/bmjdrc-2020-002035 33431602 PMC7802391

[16] Jansen T. Mind the Safety Net: Socioeconomic Inequalities in Out-of-Hours Primary Care Use. Utrecht: Nivel, Proefschrift van de Universiteit van Amsterdam (2020). p. 197.

[17] Seidu S Bodicoat DH Davies MJ Daly H Stribling B Farooqi A. Evaluating the impact of an enhanced primary care diabetes service on diabetes outcomes: a before–after study. Prim Care Diabetes. (2017) 11:171–7. 10.1016/j.pcd.2016.09.005 27745857

[18] Prince MJ Wu F Guo Y Gutierrez Robledo LM O'Donnell M Sullivan R. The burden of disease in older people and implications for health policy and practice. Lancet. (2015) 385:549–62. 10.1016/S0140-6736(14)61347-7 25468153

[19] Kroezen M Van Hoegaerden M Batenburg R. The joint action on health workforce planning and forecasting: results of a European programme to improve health workforce policies. Health Policy. (2018) 122:87–93. 10.1016/j.healthpol.2017.12.002 29241846

[20] Nivel Research Communication Center. Nivel Primary Care Database. (2024). Available online at: https://www.nivel.nl/en/our-databases-and-panels/nivel-primary-care-database (accessed June 18, 2024).

[21] Ramerman L Overbeek L. Nivel. De zorg die de huisartsenpost verleent - aard en omvang. (2023). Available online at: https://www.nivel.nl/nl/resultaten-van-onderzoek/zorg-verleend-de-eerste-lijn-aard-en-omvang/zorg-huisartsenpost (accessed August 15, 2023).

[22] Von Elm E Altman DG Egger M Pocock SJ Gøtzsche PC Vandenbroucke JP. The strengthening the reporting of observational studies in epidemiology (STROBE) statement: guidelines for reporting observational studies. J Clin Epidemiol. (2008) 61:344–9. 10.1016/j.jclinepi.2007.11.008 18313558

[23] Duineveld B Hole HM Van Werven H. NHG-Richtlijn Adequate dossiervorming met het elektronisch patiëntdossier (ADEPD). Utrecht. (2019). Available online at: https://www.nhg.org/praktijkvoering/informatisering/richtlijn-adequate-dossiervorming-epd/ (accessed June 18, 2024).

[24] Amsah N Md Isa Z Ahmad N Abdul Manaf MR. Impact of COVID-19 pandemic on healthcare utilization among patients with type 2 diabetes mellitus: a systematic review. Int J Environ Res Public Health. (2023) 20:4577. 10.3390/ijerph20054577 36901588 PMC10002238

[25] Van Grondelle SE Van Bruggen S Rauh SP Van Der Zwan M Cebrian A Seidu S. The impact of the covid-19 pandemic on diabetes care: the perspective of healthcare providers across Europe. Prim Care Diabetes. (2023) 17:141–7. 10.1016/j.pcd.2023.02.002 36822977 PMC9933343

[26] Homburg MT Berger M Berends M Meijer E Kupers T Ramerman L. Dutch GP healthcare consumption in COVID-19 heterogeneous regions: an interregional time-series approach in 2020-2021. BJGP Open. (2024) 8. 10.3399/BJGPO.2023.0121 38128964 PMC11300972

[27] van den Berg JM Blom MT Swart KMA Overbeek JA Remmelzwaal S Elders PJM. The impact of the COVID-19 pandemic in the Netherlands on primary healthcare use and clinical outcomes in persons with type 2 diabetes. COVID. (2023) 3:1677–87. 10.3390/covid3110115

[28] Bak JCG Serné EH Groenwold RHH de Valk HW Kramer MHH Nieuwdorp M. Effects of COVID-19 on diabetes care among Dutch diabetes outpatients. Diabetol Metab Syndr. (2023) 15:193. 10.1186/s13098-023-01169-9 37817214 PMC10563332

[29] Misra S Barron E Vamos E Thomas S Dhatariya K Kar P. Temporal trends in emergency admissions for diabetic ketoacidosis in people with diabetes in England before and during the COVID-19 pandemic: a population-based study. Lancet Diabetes Endocrinol. (2021) 9:671–80. 10.1016/S2213-8587(21)00208-4 34481558 PMC9765220

[30] Hartmann-Boyce J Highton P Rees K Onakpoya I Suklan J Curtis F. The impact of the COVID-19 pandemic and associated disruptions in health-care provision on clinical outcomes in people with diabetes: a systematic review. Lancet Diabetes Endocrinol. (2024) 12:132–48. 10.1016/S2213-8587(23)00351-0 38272607

[31] Stachteas P Symvoulakis M Tsapas A Smyrnakis E. The impact of the COVID-19 pandemic on the management of patients with chronic diseases in primary health care. Popul Med. (2022) 4:23. 10.18332/popmed/152606 PMC9923072 36818259

[32] Hyun MK Lee JW Ko SH. Chronic disease management program applied to type 2 diabetes patients and prevention of diabetic complications: a retrospective cohort study using nationwide data. BMC Public Health. (2023) 23:928. 10.1186/s12889-023-15763-z 37221526 PMC10203667

[33] Wanni Arachchige Dona S Angeles MR Hall N Watts JJ Peeters A Hensher M. Impacts of chronic disease prevention programs implemented by private health insurers: a systematic review. BMC Health Serv Res. (2021) 21:1222. 10.1186/s12913-021-07212-7 34763676 PMC8582197

[34] Chan KS Yuk-Fai Wan E Chin WY Ho-Gi Cheng W Kay Ho M Yee-Tak Yu E. Effects of continuity of care on health outcomes among patients with diabetes mellitus and/or hypertension: a systematic review. BMC Fam Pract. (2021) 22:145. 10.1186/s12875-021-01493-x 34217212 PMC8254900

[35] Wong VW Wang A Manoharan M. Utilisation of telehealth for outpatient diabetes management during COVID-19 pandemic: how did the patients fare? Intern Med J. (2021) 51:2021–6. 10.1111/imj.15441 34227718 PMC8447012

[36] M Tourkmani A J ALHarbi T Rsheed AMB Alrasheedy AA ALMadani W ALJuraisi F. The impact of telemedicine on patients with uncontrolled type 2 diabetes mellitus during the COVID-19 pandemic in Saudi Arabia: findings and implications. J Telemed Telecare. (2023) 29:390–8. 10.1177/1357633X20985763 33525952 PMC10195693

[37] Bouwman J Boorsma A Bouter CA Kuijper S Dulos R Best de DD. Pandemisch Paraat Door Middel van Herbruikbare Data. (2024).

[38] Rayman G Akpan A Cowie M Evans R Patel M Posporelis S. Managing patients with comorbidities: future models of care. Future Healthc J. (2022) 9:101–5. 10.7861/fhj.2022-0029 35928198 PMC9345245

[39] De Berardis G D'Ettorre A Graziano G Lucisano G Pellegrini F Cammarota S. The burden of hospitalization related to diabetes mellitus: a population-based study. Nutr Metab Cardiovasc Dis. (2012) 22:605–12. 10.1016/j.numecd.2010.10.016 21333508

[40] GDPR. Art. 9 GDPR - Processing of Special Categories of Personal Data. Available online at: https://gdpr-info.eu/art-9-gdpr/ (accessed April 25, 2024).

