# Supplementary material for: Downsizing chronic disease management programs for type 2 diabetes patients during the COVID-19 pandemic: changes in healthcare utilization patterns
Source: Front Med (Lausanne). 2025 Jun 11;12:1490175. doi: 10.3389/fmed.2025.1490175 (PMC12187798; doi:10.3389/fmed.2025.1490175)
Supplement: Supplementary file 2 [file Table_2.docx]

Supplementary Material

# Supplementary Tables

Table S1. Reimbursement codes for diabetes hospital care used in this study

| Reimbursement codes | Description |
| --- | --- |
| Examination(s)/treatment(s) diabetes | 040201004; 040201009 |
| Hospital admission diabetes | 040201006; 040201007; 040201019; 040201013; 040201014; 040201016 |
| Outpatients clinic visits/remote consultations diabetes | 040201008; 040201010; 040201011 |
| Refilling internal insulin pump for diabetes | 040201005; 040201020 |
| Placing a sensor (RT-CGM) for diabetes | 040201021 |
| Operation to remove the vitreous in retinal disease caused by diabetes | 079799003 |
| Treatment(s)/nursing days for retinal disease caused by diabetes | 079799006 |
| Injection of medication into the vitreous for retinal disease caused by diabetes | 079799007 |
| Outpatients clinic visits/remote consultations with diagnostic testing for retinal disease caused by diabetes | 079799012; 079799022; 079799024; 079799031; 079799033 |
| Outpatients clinic visits/remote consultations for retinal disease caused by diabetes | 079799023; 079799032; 079799036 |
| Laser treatment of retinal abnormalities in retinal disease caused by diabetes | 079799025; 079799034 |
| Outpatient clinic visits/remote consultations for an eye infection, disease of the retina/choroid/vitreous body in the eye, no retinal disease due to diabetes | 079799037 |
| Surgery for retinal disease caused by diabetes | 079799038; 079799041 |
| Surgical repair of a retinal detachment in retinal disease caused by diabetes | 079799045 |
| Treatment(s)/nursing days for retinal disease caused by diabetes | 079799048; 079799013; 079799014 |
| Treatment(s)/nursing days for a foot abnormality as a result of diabetes | 099699007; 099699020; 099699021 |
| Examination in the vascular laboratory for an abnormality of the foot due to diabetes | 099699018; 099699019 |
| (Bone) surgery and/or amputation for a foot abnormality due to diabetes | 099699022; 099699023; 099699059; 099699060; 099699063; 099699064 |
| Examination(s) and/or treatment(s) for a foot abnormality due to diabetes | 099699034; 099699035; 099699036 |
| Outpatient clinic visits/remote consultations for a foot abnormality due to diabetes | 099699058; 099699076; 099699083 |
| Hospital admission by surgeon in case of kidney failure or diabetes | 140301014; 140301035; 140301036 |
| Implanting or replacing an insulin pump for diabetes | 140301015; 140301016 |
| Diagnostic examination by surgeon in case of kidney failure or diabetes | 140301034 |
| Surgery for kidney failure or diabetes | 140301038; 140301039 |
| Treatment(s) and/or outpatient clinic visits/remote consultations and/or examination(s) (by a surgeon) for kidney failure or diabetes | 140301055; 140301062; 140301080 |
| Treatment at the outpatient clinic with Insulin pump or shunt or port-a-cath | 140301013 |
| Diabetes, other | 040201001; 040201012; 040201015; 079799046 |

From: The Dutch Healthcare Authority. Zorgproducten. 2024. Available from: https://zorgproducten.nza.nl/ZoekZorgproduct.aspx [Last accessed: 7/2/2024].

Table S2. Explanation of the variables.

| Variable | Explanation | Data sources |
| --- | --- | --- |
| Gender | 1. Male  2. Female | Nivel-PCD |
| Age | Age categories:  1. 18-44  2. 45-64  3. 65-74  4. 75-84  5. 85 years and older | CBS Microdata |
| Migration background | 1. Dutch Background  2. Western background (countries in Europe (excluding Turkey), North America, Oceania, Indonesia or Japan) 3. Non-western background (countries in Africa, Latin America, and Asia (excluding Indonesia, Japan), and Turkey)  Note: an individual has a Western or non-Western migration background if born abroad or if at least one of their parents was born abroad. | CBS Microdata |
| Standardized household income | 1. Low household income (percentile 1-39) 2. Middle household income (percentile 40-79) 3. High household income (percentile 80-100) | CBS Microdata |

Table S3a. Number of contact rates per 1,000 patients in 2019, 2020 and 2021, and differences in contact rates between (2019 and 2020) and (2019 and 2021), per sub-group for age and migration background

|  |  | 2019  Number of contacts per 1,000 patients | | | | 2020  Number of contacts per 1,000 patients  (difference compared to 2019) | | | | 2021  Number of contacts per 1,000 patients  (difference compared to 2019) | | | | |
| --- | --- | --- | --- | --- | --- | --- | --- | --- | --- | --- | --- | --- | --- | --- |
|  |  | Q1 | Q2 | Q3 | Q4 | Q1 | Q2 | Q3 | Q4 | Q1 | Q2 | Q3 | Q4 |  |
| 65 years and older | Diabetes care programs at the GP | 629 | 570 | 580 | 567 | 494 (-21.4%) | 384 (-32.6%) | 500 (-13.8%) | 433 (-23.7%) | 443 (-29.6%) | 423 (-25.8%) | 389 (-33.0%) | 394 (-30.5%) |  |
|  | Regular general practice care | 2.099 | 1.969 | 1.998 | 2.011 | 2.056 (-2.0%) | 1.845 (-6.3%) | 2.063 (3.2%) | 2.113 (5.1%) | 2.261 (7.7%) | 2.236 (13.6%) | 2.188 (9.5%) | 2.335 (16.1%) |  |
|  | Hospital care | 37 | 36 | 34 | 36 | 32 (-13.6%) | 27 (-25.2%) | 35 (2.6%) | 36 (-2.3%) | 36 (-2.1%) | 37 (3.0%) | 35 (2.7%) | 36 (-1.8%) |  |
| 18-64 years | Diabetes care programs at the GP | 602 | 568 | 568 | 575 | 492 (-18.2%) | 345 (-39.2%) | 439 (-22.8%) | 418 (-27.3%) | 389 (-35.3%) | 375 (-34.0%) | 332 (-41.6%) | 355 (-38.2%) |  |
|  | Regular general practice care | 1.815 | 1.663 | 1.674 | 1.693 | 1.756 (-3.3%) | 1.480 (-11.0%) | 1.578 (-5.7%) | 1.646 (-2.8%) | 1.733 (-4.5%) | 1.855 (11.5%) | 1.596 (-4.6%) | 1.674 (-1.1%) |  |
|  | Hospital care | 37 | 38 | 40 | 43 | 43 (17.6%) | 36 (-4.8%) | 39 (-2.3%) | 45 (4.1%) | 53 (44.0%) | 50 (30.1%) | 47 (16.8%) | 48 (11.7%) |  |
|  |  |  |  |  |  |  |  |  |  |  |  |  |  |  |
| Dutch background | Diabetes care programs at the GP | 613 | 567 | 585 | 579 | 495 (-19.2%) | 392 (-30.8%) | 495 (-15.4%) | 446 (-23.0%) | 464 (-24.2%) | 449 (-20.9%) | 411 (-29.8%) | 424 (-26.8%) |  |
|  | Regular general practice care | 2.001 | 1.871 | 1.932 | 1.905 | 1.955 (-2.3%) | 1.765 (-5.7%) | 1.934 (0.1%) | 1.977 (3.8%) | 2.137 (6.8%) | 2.123 (13.5%) | 2.083 (7.8%) | 2.186 (14.7%) |  |
|  | Hospital care | 33 | 32 | 31 | 33 | 30 (-10.3%) | 25 (-19.9%) | 33 (5.9%) | 34 (4.1%) | 36 (7.9%) | 36 (12.8%) | 33 (7.6%) | 34 (2.2%) |  |
| Migration background | Diabetes care programs at the GP | 633 | 575 | 550 | 547 | 488 (-23.0%) | 312 (-45.7%) | 434 (-21.0%) | 379 (-30.7%) | 322 (-49.2%) | 297 (-48.3%) | 263 (-52.2%) | 270 (-50.7%) |  |
|  | Regular general practice care | 1.964 | 1.804 | 1.723 | 1.849 | 1.940 (-1.2%) | 1.591 (-11.8%) | 1.785 (3.6%) | 1.875 (1.4%) | 1.969 (0.3%) | 2.089 (15.8%) | 1.769 (2.7%) | 1.954 (5.7%) |  |
|  | Hospital care | 46 | 50 | 51 | 56 | 56 (12.0%) | 42 (-14.6%) | 47 (-8.9%) | 52 (-7.4%) | 56 (22.0%) | 55 (9.7%) | 54 (5.2%) | 57 (1.7%) |  |

Table S3b. Number of contact rates per 1,000 patients in 2019, 2020 and 2021, and differences in contact rates between (2019 and 2020) and (2019 and 2021), per sub-group for household income

|  |  | | | 2019  Number of contacts per 1,000 patients | | | 2020  Number of contacts per 1,000 patients  (difference compared to 2019) | | | | 2021  Number of contacts per 1,000 patients  (difference compared to 2019) | | | |
| --- | --- | --- | --- | --- | --- | --- | --- | --- | --- | --- | --- | --- | --- | --- |
|  |  | Q1 | Q2 | | Q3 | Q4 | Q1 | Q2 | Q3 | Q4 | Q1 | Q2 | Q3 | Q4 |
| Low income | Diabetes care programs at the GP | 630 | 576 | | 583 | 566 | 499 (-20.8%) | 376 (-34.7%) | 479 (-17.8%) | 423 (-25.3%) | 410 (-35.0%) | 400 (-30.6%) | 360 (-38.2%) | 371 (-34.5%) |
|  | Regular general practice care | 2.220 | 2.031 | | 2.111 | 2.112 | 2.170 (-2.2%) | 1.934 (-4.8%) | 2.127 (0.7%) | 2.177 (3.1%) | 2.341 (5.5%) | 2.339 (15.2%) | 2.230 (5.6%) | 2.360 (11.8%) |
|  | Hospital care | 40 | 39 | | 37 | 45 | 39 (-2.3%) | 33 (-13.6%) | 38 (2.3%) | 39 (-13.3%) | 40 (1.7%) | 42 (8.6%) | 39 (6.0%) | 41 (-8.6%) |
| Middle income | Diabetes care programs at the GP | 613 | 567 | | 583 | 572 | 496 (-19.1%) | 376 (-33.7%) | 491 (-15.8%) | 441 (-22.9%) | 451 (-26.5%) | 426 (-24.8%) | 393 (-32.6%) | 400 (-30.0%) |
|  | Regular general practice care | 1.829 | 1.760 | | 1.723 | 1.712 | 1.772 (-3.1%) | 1.513 (-14.0%) | 1.723 (0.0%) | 1.735 (1.4%) | 1.850 (1.1%) | 1.916 (8.9%) | 1.799 (4.4%) | 1.891 (10.4%) |
|  | Hospital care | 30 | 32 | | 35 | 34 | 31 (2.7%) | 24 (-25.4%) | 34 (-3.6%) | 40 (17.6%) | 40 (33.3%) | 40 (24.5%) | 37 (5.5%) | 36 (7.2%) |
| High income | Diabetes care programs at the GP | 588 | 548 | | 524 | 581 | 468 (-20.4%) | 339 (-38.1%) | 445 (-15.1%) | 408 (-29.9%) | 424 (-27.8%) | 390 (-28.9%) | 354 (-32.5%) | 380 (-34.7%) |
|  | Regular general practice care | 1.527 | 1.370 | | 1.350 | 1.472 | 1.462 (-4.2%) | 1.274 (-7.0%) | 1.326 (-1.8%) | 1.516 (3.0%) | 1.477 (-3.2%) | 1.511 (10.3%) | 1.375 (1.9%) | 1.538 (4.5%) |
|  | Hospital care | 44 | 38 | | 36 | 31 | 36 (-17.9%) | 34 (-12.6%) | 38 (6.1%) | 37 (22.2%) | 52 (18.9%) | 37 (-4.2%) | 46 (29.6%) | 48 (55.6%) |
